# Supplementary material for: Design of a BIST implemented AES crypto-processor ASIC
Source: PLoS One. 2021 Nov 16;16(11):e0259956. doi: 10.1371/journal.pone.0259956 (PMC8594793; doi:10.1371/journal.pone.0259956)
Supplement: S1 Appendix — Controller module to all other modules are presented in the appendix. (DOCX) [file pone.0259956.s001.docx]

**APPENDIX A**

**CONTROLLER MODULE**

**// Controller Module**

module Controller(input wire[127:0] keyI, input wire[127:0] initialState, input wire normalEncryption, input wire normalDecryption, input wire decryptionFollowsEncryption, input wire encryptionForRandom, input wire decryptionForRandom, input wire bistMode, input wire clk,

input wire rst, output reg[127:0] plainTextValue,output reg[127:0] cipherTextValue,output reg[31:0] matchedSignature,output reg result

);

reg [127:0] key_byte,valueI,oraI;

wire [127:0] state_byte;

reg ecryptionEnable,enable,decryptionEnable,decryptionSecondEnable,oraEnable,startPatternGeneration;

wire [127:0] state_out_dec,state_out_enc,state_second_dec;

wire [31:0] state_out_ora;

wire load,readyE,readyD,resultO,readyK,readyOra;

integer i,j;

reg enableEncryption,enableDecryption;

reg flag=0;

reg [31:0] temp[0:21];

reg[31:0] generatePattern [0:3];

integer iterate;

reg [31:0] encryptionSignature=32'hBC52CEBF; //Golden signature for encryption

reg [31:0] decryptionSignature=32'h6081AF79; //Golden signature for decryption

AES_encryption AES_TB(key_byte,valueI,clk,rst,ecryptionEnable,state_out_enc,load,readyE);

AES_decryption SEC_TB(key_byte,state_out_enc,clk,rst,decryptionSecondEnable,state_second_dec,load,readyD);

AES_decryption DEC_TB(key_byte,valueI,clk,rst,decryptionEnable,state_out_dec,load,readyK);

ORA o(clk,rst,oraEnable,oraI,state_out_ora,readyOra);

always@(posedge clk)begin : break_block

if(j>=62)begin

disable break_block;

end

if(rst)begin

plainTextValue = 128'h00000000000000000000000000000000;

cipherTextValue = 128'h00000000000000000000000000000000;

end

if(flag==0)begin

startPatternGeneration=1;

j=0;

iterate=0;

temp[0]=32'b11111111111111111111111111111111; /* Default seed value to generate random values */

temp[1]=32'b00000000000000000000000000011111; /* 21 seed values (temp[1] to temp[21]) stored in the memory to generate deterministic patterns */

temp[2]=32'b00000000000000000000000000001111;

temp[3]=32'b00000000000000000000000000000111;

temp[4]=32'b00000000000000000000000000000011;

temp[5]=32'b00000000000000000000000000000001;

temp[6]=32'b00000000000000000000001111100000;

temp[7]=32'b00000000000000000000001111000000;

temp[8]=32'b00000000000000000000001110000000;

temp[9]=32'b00000000000000000000001100000000;

temp[10]=32'b00000000000000000000001000000000;

temp[11]=32'b00000000000000000000001000000001;

temp[12]=32'b00000000000000000000001100000011;

temp[13]=32'b00000000000000000000001110000001;

temp[14]=32'b00000000000000000000001111000001;

temp[15]=32'b00000000000000000000001111100001;

temp[16]=32'b00000000000000000000001111110001;

temp[17]=32'b00000000000000000000001111111001;

temp[18]=32'b00000000000000000000001111111101;

temp[19]=32'b00000000000000000000000000000001;

temp[20]=32'b00000000000000000000000000000010;

temp[21]=32'b00000000000000000000000000000100;

flag=1;

end

if((normalEncryption===1) && (bistMode===0))begin //Encryption starts

ecryptionEnable = 1;

valueI = initialState;

key_byte = keyI;

cipherTextValue = state_out_enc;

end //End of encryption

else if((normalDecryption===1) && (bistMode===0))begin //Decryption starts

decryptionEnable = 1;

valueI = initialState;

key_byte = keyI;

plainTextValue = state_out_dec;

end //End of decryption

else if((decryptionFollowsEncryption===1) && (bistMode===0))begin //Decryption following Encryption

ecryptionEnable = 1;

valueI = initialState;

key_byte=keyI;

cipherTextValue = state_out_enc;

decryptionSecondEnable = 1;

plainTextValue = state_second_dec;

end //End after obtaining the actual plain text value

else if(((decryptionForRandom===1) && (bistMode===1)) || ((encryptionForRandom===1) && (bistMode===1)))begin

//Enables the test mode either for encryption or decryption

if(startPatternGeneration==1)begin

for(i=0;i<4;i=i+1)begin /*Linear-feedback shift register (LFSR) to generate test patterns */

temp[iterate]={(temp[iterate][31] ^ temp[iterate][25] ^ temp[iterate][22] ^ temp[iterate][21] ^ temp[iterate][15] ^ temp[iterate][11] ^ temp[iterate][10] ^ temp[iterate][9] ^ temp[iterate][7] ^ temp[iterate][6] ^ temp[iterate][4] ^ temp[iterate][3] ^ temp[iterate][1] ^ temp[iterate][0]), temp[iterate][31:1]};

generatePattern[i]=temp[iterate];

end

end

if(encryptionForRandom)

ecryptionEnable=1;

else

decryptionEnable=1;

startPatternGeneration=0;

valueI = {generatePattern[3],generatePattern[2],generatePattern[1],generatePattern[0]};

key_byte=keyI;

oraEnable=0;

if((readyE==1) || (readyK==1))begin

if(j>=39)begin

iterate=iterate+1;

end

oraEnable=1;

oraI = encryptionForRandom ? (ecryptionEnable ? state_out_enc : 128'h00000000000000000000000000000000) : (decryptionEnable ? state_out_dec : 128'h00000000000000000000000000000000);

if(encryptionForRandom)

ecryptionEnable=0;

else

decryptionEnable=0;

matchedSignature = state_out_ora;

if((matchedSignature==encryptionSignature) || (matchedSignature==decryptionSignature))begin

result=1; // Correct matching of golden signature and candidate signature

j=j+1;

end

else begin

result=0; // Mismatch of golden signature and candidate signature

end

startPatternGeneration=1;

j=j+1;

end

end

end //End always

endmodule

**ENCRYPTION MODULE**

**// AES_encryption Module**

module AES_encryption

(

input [127:0] byteKey, byteText,

input clk,rst,enable,

output reg [127:0] outputResult,

output reg fetchAll,initialStep

);

integer i,j,y;

reg [1:0] loop1;

reg [1:0] loop2;

reg [127:0] key, text;

reg start,startRotate,startColumnMixing,startYK;

wire done,endRotating,endColumnMixing;

reg finish;

reg [127:0] sendTextInput,receiveTextOutput;

reg [127:0] sendKeyInput;

wire [127:0] roundValueO,subBytesValueO,shiftRowsValueO;

wire [127:0] mixColumnsValueO;

reg [3:0] keyNum;

reg taskComplete;

reg [127:0] kTransferO [10:0];

reg [7:0] a1;

reg [7:0] a2;

reg [7:0] a3;

reg [7:0] a4;

reg [7:0] c;

reg [31:0] rcon [10:0];

reg [127:0] keyInputI;

reg [127:0] keyOuputO;

reg [31:0] ladder1 ;

reg [31:0] ladder2 ;

task getResult;

input [7:0] z;

case (z) // Sbox values for byte substitution during key expansion

8'h00: c=8'h63;

8'h01: c=8'h7c;

8'h02: c=8'h77;

8'h03: c=8'h7b;

8'h04: c=8'hf2;

8'h05: c=8'h6b;

8'h06: c=8'h6f;

8'h07: c=8'hc5;

8'h08: c=8'h30;

8'h09: c=8'h01;

8'h0a: c=8'h67;

8'h0b: c=8'h2b;

8'h0c: c=8'hfe;

8'h0d: c=8'hd7;

8'h0e: c=8'hab;

8'h0f: c=8'h76;

8'h10: c=8'hca;

8'h11: c=8'h82;

8'h12: c=8'hc9;

8'h13: c=8'h7d;

8'h14: c=8'hfa;

8'h15: c=8'h59;

8'h16: c=8'h47;

8'h17: c=8'hf0;

8'h18: c=8'had;

8'h19: c=8'hd4;

8'h1a: c=8'ha2;

8'h1b: c=8'haf;

8'h1c: c=8'h9c;

8'h1d: c=8'ha4;

8'h1e: c=8'h72;

8'h1f: c=8'hc0;

8'h20: c=8'hb7;

8'h21: c=8'hfd;

8'h22: c=8'h93;

8'h23: c=8'h26;

8'h24: c=8'h36;

8'h25: c=8'h3f;

8'h26: c=8'hf7;

8'h27: c=8'hcc;

8'h28: c=8'h34;

8'h29: c=8'ha5;

8'h2a: c=8'he5;

8'h2b: c=8'hf1;

8'h2c: c=8'h71;

8'h2d: c=8'hd8;

8'h2e: c=8'h31;

8'h2f: c=8'h15;

8'h30: c=8'h04;

8'h31: c=8'hc7;

8'h32: c=8'h23;

8'h33: c=8'hc3;

8'h34: c=8'h18;

8'h35: c=8'h96;

8'h36: c=8'h05;

8'h37: c=8'h9a;

8'h38: c=8'h07;

8'h39: c=8'h12;

8'h3a: c=8'h80;

8'h3b: c=8'he2;

8'h3c: c=8'heb;

8'h3d: c=8'h27;

8'h3e: c=8'hb2;

8'h3f: c=8'h75;

8'h40: c=8'h09;

8'h41: c=8'h83;

8'h42: c=8'h2c;

8'h43: c=8'h1a;

8'h44: c=8'h1b;

8'h45: c=8'h6e;

8'h46: c=8'h5a;

8'h47: c=8'ha0;

8'h48: c=8'h52;

8'h49: c=8'h3b;

8'h4a: c=8'hd6;

8'h4b: c=8'hb3;

8'h4c: c=8'h29;

8'h4d: c=8'he3;

8'h4e: c=8'h2f;

8'h4f: c=8'h84;

8'h50: c=8'h53;

8'h51: c=8'hd1;

8'h52: c=8'h00;

8'h53: c=8'hed;

8'h54: c=8'h20;

8'h55: c=8'hfc;

8'h56: c=8'hb1;

8'h57: c=8'h5b;

8'h58: c=8'h6a;

8'h59: c=8'hcb;

8'h5a: c=8'hbe;

8'h5b: c=8'h39;

8'h5c: c=8'h4a;

8'h5d: c=8'h4c;

8'h5e: c=8'h58;

8'h5f: c=8'hcf;

8'h60: c=8'hd0;

8'h61: c=8'hef;

8'h62: c=8'haa;

8'h63: c=8'hfb;

8'h64: c=8'h43;

8'h65: c=8'h4d;

8'h66: c=8'h33;

8'h67: c=8'h85;

8'h68: c=8'h45;

8'h69: c=8'hf9;

8'h6a: c=8'h02;

8'h6b: c=8'h7f;

8'h6c: c=8'h50;

8'h6d: c=8'h3c;

8'h6e: c=8'h9f;

8'h6f: c=8'ha8;

8'h70: c=8'h51;

8'h71: c=8'ha3;

8'h72: c=8'h40;

8'h73: c=8'h8f;

8'h74: c=8'h92;

8'h75: c=8'h9d;

8'h76: c=8'h38;

8'h77: c=8'hf5;

8'h78: c=8'hbc;

8'h79: c=8'hb6;

8'h7a: c=8'hda;

8'h7b: c=8'h21;

8'h7c: c=8'h10;

8'h7d: c=8'hff;

8'h7e: c=8'hf3;

8'h7f: c=8'hd2;

8'h80: c=8'hcd;

8'h81: c=8'h0c;

8'h82: c=8'h13;

8'h83: c=8'hec;

8'h84: c=8'h5f;

8'h85: c=8'h97;

8'h86: c=8'h44;

8'h87: c=8'h17;

8'h88: c=8'hc4;

8'h89: c=8'ha7;

8'h8a: c=8'h7e;

8'h8b: c=8'h3d;

8'h8c: c=8'h64;

8'h8d: c=8'h5d;

8'h8e: c=8'h19;

8'h8f: c=8'h73;

8'h90: c=8'h60;

8'h91: c=8'h81;

8'h92: c=8'h4f;

8'h93: c=8'hdc;

8'h94: c=8'h22;

8'h95: c=8'h2a;

8'h96: c=8'h90;

8'h97: c=8'h88;

8'h98: c=8'h46;

8'h99: c=8'hee;

8'h9a: c=8'hb8;

8'h9b: c=8'h14;

8'h9c: c=8'hde;

8'h9d: c=8'h5e;

8'h9e: c=8'h0b;

8'h9f: c=8'hdb;

8'ha0: c=8'he0;

8'ha1: c=8'h32;

8'ha2: c=8'h3a;

8'ha3: c=8'h0a;

8'ha4: c=8'h49;

8'ha5: c=8'h06;

8'ha6: c=8'h24;

8'ha7: c=8'h5c;

8'ha8: c=8'hc2;

8'ha9: c=8'hd3;

8'haa: c=8'hac;

8'hab: c=8'h62;

8'hac: c=8'h91;

8'had: c=8'h95;

8'hae: c=8'he4;

8'haf: c=8'h79;

8'hb0: c=8'he7;

8'hb1: c=8'hc8;

8'hb2: c=8'h37;

8'hb3: c=8'h6d;

8'hb4: c=8'h8d;

8'hb5: c=8'hd5;

8'hb6: c=8'h4e;

8'hb7: c=8'ha9;

8'hb8: c=8'h6c;

8'hb9: c=8'h56;

8'hba: c=8'hf4;

8'hbb: c=8'hea;

8'hbc: c=8'h65;

8'hbd: c=8'h7a;

8'hbe: c=8'hae;

8'hbf: c=8'h08;

8'hc0: c=8'hba;

8'hc1: c=8'h78;

8'hc2: c=8'h25;

8'hc3: c=8'h2e;

8'hc4: c=8'h1c;

8'hc5: c=8'ha6;

8'hc6: c=8'hb4;

8'hc7: c=8'hc6;

8'hc8: c=8'he8;

8'hc9: c=8'hdd;

8'hca: c=8'h74;

8'hcb: c=8'h1f;

8'hcc: c=8'h4b;

8'hcd: c=8'hbd;

8'hce: c=8'h8b;

8'hcf: c=8'h8a;

8'hd0: c=8'h70;

8'hd1: c=8'h3e;

8'hd2: c=8'hb5;

8'hd3: c=8'h66;

8'hd4: c=8'h48;

8'hd5: c=8'h03;

8'hd6: c=8'hf6;

8'hd7: c=8'h0e;

8'hd8: c=8'h61;

8'hd9: c=8'h35;

8'hda: c=8'h57;

8'hdb: c=8'hb9;

8'hdc: c=8'h86;

8'hdd: c=8'hc1;

8'hde: c=8'h1d;

8'hdf: c=8'h9e;

8'he0: c=8'he1;

8'he1: c=8'hf8;

8'he2: c=8'h98;

8'he3: c=8'h11;

8'he4: c=8'h69;

8'he5: c=8'hd9;

8'he6: c=8'h8e;

8'he7: c=8'h94;

8'he8: c=8'h9b;

8'he9: c=8'h1e;

8'hea: c=8'h87;

8'heb: c=8'he9;

8'hec: c=8'hce;

8'hed: c=8'h55;

8'hee: c=8'h28;

8'hef: c=8'hdf;

8'hf0: c=8'h8c;

8'hf1: c=8'ha1;

8'hf2: c=8'h89;

8'hf3: c=8'h0d;

8'hf4: c=8'hbf;

8'hf5: c=8'he6;

8'hf6: c=8'h42;

8'hf7: c=8'h68;

8'hf8: c=8'h41;

8'hf9: c=8'h99;

8'hfa: c=8'h2d;

8'hfb: c=8'h0f;

8'hfc: c=8'hb0;

8'hfd: c=8'h54;

8'hfe: c=8'hbb;

8'hff: c=8'h16;

endcase

endtask

AddRoundKey S(.inputkey(sendKeyInput),.inputState(sendTextInput),.clk(clk),.reset(rst),.enable(start),.inputOut(roundValueO),.success(done));

genvar itr;

generate

for (itr = 0 ; itr <= 127; itr = itr+32) begin : block_subbyte

SubBytes statSub (.valueI(roundValueO[itr +:32]) , .valueO(subBytesValueO[itr +:32]));

end

endgenerate

ShiftRows Sft (.shiftEnable(startRotate),.clk(clk),.reset(rst),.value(subBytesValueO),.valueShifted(shiftRowsValueO),.success(endRotating) );

MixColumns M (.value(sendTextInput),.clk (clk),.enableMixColumn(startColumnMixing), .reset(rst),.valueOut(mixColumnsValueO),.success(endColumnMixing));

always @(posedge clk)begin

if (rst) begin

taskComplete <= 0;

key<=128'd0;

text<=128'd0;

fetchAll<=1'd0;

initialStep<=1'd0;

i <= 1;

outputResult <= 128'b00000000000000000000000000000000000000000000000000000000000000000000000000000000000000000000000000000000000000000000000000000000;

sendTextInput <= 128'b00000000000000000000000000000000000000000000000000000000000000000000000000000000000000000000000000000000000000000000000000000000;

receiveTextOutput <= 128'b00000000000000000000000000000000000000000000000000000000000000000000000000000000000000000000000000000000000000000000000000000000;

j <= 1;

loop2 <= 0 ;

end

else if (enable) begin

i<=1;

case(loop2)

0:

begin

if(i>0)begin

key<=byteKey; // Initializewith the input key

text<=byteText; // Initialize with the input text

taskComplete<=1;

i<=i-1;

end

if(finish)begin

initialStep <= 1;

outputResult <= receiveTextOutput; // Resultant ciphertext

end

else begin

initialStep <= 0;

end

end

endcase

end else loop2 <= 0;

if (rst)

begin

keyNum <= 0;

loop1 <= 0;

startColumnMixing <= 0;

startYK <= 0;

start <= 0;

startRotate <= 0;

receiveTextOutput <= 0;

finish <= 0;

end

else if( (enable == 1 ) && (taskComplete == 1))

begin

if ( keyNum <= 11 )

begin

case (loop1)

2'b00:

begin

// Start of key expansion

kTransferO[0] = byteKey;

kTransferO[1] = 128'b0;

kTransferO[2] = 128'b0;

kTransferO[3] = 128'b0;

kTransferO[4] = 128'b0;

kTransferO[5] = 128'b0;

kTransferO[6] = 128'b0;

kTransferO[7] = 128'b0;

kTransferO[8] = 128'b0;

kTransferO[9] = 128'b0;

kTransferO[10] = 128'b0;

rcon[1] = 32'h01000000; // Contant values to generate round keys

rcon[2] = 32'h02000000;

rcon[3] = 32'h04000000;

rcon[4] = 32'h08000000;

rcon[5] = 32'h10000000;

rcon[6] = 32'h20000000;

rcon[7] = 32'h40000000;

rcon[8] = 32'h80000000;

rcon[9] = 32'h1b000000;

rcon[10] = 32'h36000000;

for (y = 1; y <= 10; y = y + 1) begin

keyOuputO = 0;

keyInputI = kTransferO[y-1];

ladder1 = { keyInputI[23:0] , keyInputI[31:24] };

getResult(ladder1[23:16]);

a1 = c;

getResult(ladder1[15:8]);

a2 = c;

getResult(ladder1[7:0]);

a3 = c;

getResult(ladder1[31:24]);

a4 = c;

ladder2 = { a4, a1, a2, a3 };

keyOuputO[127:96] = keyInputI[127:96] ^ ladder2 ^ rcon[y];

keyOuputO[95:64] = keyInputI[95:64] ^ keyOuputO[127:96];

keyOuputO[63:32] = keyInputI[63:32] ^ keyOuputO[95:64];

keyOuputO[31:0] = keyInputI[31:0] ^ keyOuputO[63:32];

kTransferO[y] = keyOuputO;

end // End of key expansion

sendKeyInput <= kTransferO[keyNum];

sendTextInput <= text;

keyNum <= 4'h1;

start <= 1;

loop1 <= 2'b01;

finish <= 0;

end

2'b01:

begin

// round shiftrows

if (done == 1)

begin

sendTextInput <= roundValueO;

sendKeyInput <= kTransferO[keyNum];

start <= 0;

startRotate <= 1;

if ( keyNum < 10) begin

loop1 <= 2'b10;

end

else

begin

loop1 <= 2'b11;

end

end

finish <= 0;

end

2'b10:

begin

// round mix columns

if (endRotating == 1)

begin

sendTextInput <= shiftRowsValueO;

startRotate <= 0;

startColumnMixing <= 1;

loop1 <= 2'b11;

end

finish <= 0;

end

2'b11:

begin

// Add round key

if (endColumnMixing == 1 || (endRotating == 1 && keyNum >= 10))

begin

if(keyNum < 10) sendTextInput <= mixColumnsValueO;

else sendTextInput <= shiftRowsValueO;

startColumnMixing <= 0;

startRotate <= 0;

start <= 1;

loop1 <= 2'b01;

keyNum <= keyNum + 1;

end

finish <= 0;

end

endcase

end

else

begin

// When keyNum > 11

initialStep<=0;

receiveTextOutput <= roundValueO;

finish <= 1;

keyNum <= 0;

loop1 <= 0;

startColumnMixing <= 0;

startYK <= 0;

start <= 0;

startRotate <= 0;

end

end else

begin

keyNum <= 0;

loop1 <= 0;

startColumnMixing <= 0;

startYK <= 0;

start <= 0;

startRotate <= 0;

receiveTextOutput <= 0;

finish <= 0;

end

end //End always

endmodule

**ADD ROUND KEY MODULE**

**// AddRoundKey Module**

module AddRoundKey(

input wire[127:0] inputkey,

input wire[127:0] inputState,

input clk,enable,reset,

output reg[127:0] inputOut,

output reg success);

integer i;

always@(posedge clk)

begin

if(reset)begin

inputOut<=128'h00000000000000000000000000000000;

success<=0;

i<=0;

end

else if(enable) begin // Input text exclusive ored with the round key

for ( i=0; i<=15; i=i+1)

inputOut[i*8 +: 8] <= inputkey[i*8 +: 8] ^ inputState[i*8 +: 8];

success<=1;

end // End of XOR

else success<=0;

end //End always

endmodule

**SUBSTITUTION BYTE MODULE**

**// SubBytes Module**

module SubBytes

(

input wire [31:0] valueI,

output wire [31:0]valueO

);

genvar i;

generate

for (i = 0 ; i <= 31; i = i+8) begin : break_block

sbox s (valueI[i +:8] , valueO[i +:8]);

end

endgenerate

endmodule

**SHIFT ROW MODULE**

**// ShiftRows Module**

module ShiftRows(

input shiftEnable,clk,reset,

input wire[0:127] value,

output reg[0:127] valueShifted,

output reg success);

always @(negedge clk)begin

if(reset)begin

valueShifted<=128'h00000000000000000000000000000000;

success<=0;

end

else if (shiftEnable) begin

//No shifting

valueShifted[0+:8] <= value[0+:8];

valueShifted[32+:8] <= value[32+:8];

valueShifted[64+:8] <= value[64+:8];

valueShifted[96+:8] <= value[96+:8];

//1 byte left shift

valueShifted [8+:8] <= value[40+:8];

valueShifted [40+:8] <= value[72+:8];

valueShifted [72+:8] <= value[104+:8];

valueShifted [104+:8] <= value[8+:8];

//2 byte left shift

valueShifted [16+:8] <= value[80+:8];

valueShifted [48+:8] <= value[112+:8];

valueShifted [80+:8] <= value[16+:8];

valueShifted [112+:8] <= value[48+:8];

//3 byte left shift

valueShifted [24+:8] <= value[120+:8];

valueShifted [56+:8] <= value[24+:8];

valueShifted [88+:8] <= value[56+:8];

valueShifted [120+:8] <= value[88+:8];

success <= 1;

end

else success <= 0;

end //End always

endmodule

**MIX COLUMN MODULE**

**// MixColumns Module**

module MixColumns(

input wire[127:0] value,

input clk,enableMixColumn,reset,

output reg[127:0] valueOut,

output reg success);

function [7:0] TwoMultiplied;

input [7:0] i;

begin

if(i[7] == 1) TwoMultiplied = ((i << 1) ^ 8'h1b);

else TwoMultiplied = i << 1;

end

endfunction

function [7:0] ThreeMultiplied;

input [7:0] i;

begin

ThreeMultiplied = TwoMultiplied(i) ^ i;

end

endfunction

integer j;

always@(negedge clk)

begin

if (reset)

begin

valueOut<=128'h00000000000000000000000000000000;

j<= 0;

success <= 0;

end

else if (enableMixColumn)

begin

for(j=0;j<=3;j=j+1)

begin

valueOut[j*32+:8] <= TwoMultiplied(value[(j*32)+:8])^(value[(j*32 + 8)+:8])^(value[(j*32 + 16)+:8])^ThreeMultiplied(value[(j*32 + 24)+:8]);

valueOut[(j*32 + 8)+:8] <= ThreeMultiplied(value[(j*32)+:8])^TwoMultiplied(value[(j*32 + 8)+:8])^(value[(j*32 + 16)+:8])^(value[(j*32 + 24)+:8]);

valueOut[(j*32 + 16)+:8] <= (value[(j*32)+:8])^ThreeMultiplied(value[(j*32 + 8)+:8])^TwoMultiplied(value[(j*32 + 16)+:8])^(value[(j*32 + 24)+:8]);

valueOut[(j*32 + 24)+:8] <= (value[(j*32)+:8])^(value[(j*32 + 8)+:8])^ThreeMultiplied(value[(j*32 + 16)+:8])^TwoMultiplied(value[(j*32 + 24)+:8]);

end

success <= 1;

end else success <= 0;

end //End always

endmodule

**SBOX MODULE**

**// sbox Module**

module sbox(input [7:0] a,output reg [7:0] c);

always @ ( a )

case (a)

8'h00: c=8'h63;

8'h01: c=8'h7c;

8'h02: c=8'h77;

8'h03: c=8'h7b;

8'h04: c=8'hf2;

8'h05: c=8'h6b;

8'h06: c=8'h6f;

8'h07: c=8'hc5;

8'h08: c=8'h30;

8'h09: c=8'h01;

8'h0a: c=8'h67;

8'h0b: c=8'h2b;

8'h0c: c=8'hfe;

8'h0d: c=8'hd7;

8'h0e: c=8'hab;

8'h0f: c=8'h76;

8'h10: c=8'hca;

8'h11: c=8'h82;

8'h12: c=8'hc9;

8'h13: c=8'h7d;

8'h14: c=8'hfa;

8'h15: c=8'h59;

8'h16: c=8'h47;

8'h17: c=8'hf0;

8'h18: c=8'had;

8'h19: c=8'hd4;

8'h1a: c=8'ha2;

8'h1b: c=8'haf;

8'h1c: c=8'h9c;

8'h1d: c=8'ha4;

8'h1e: c=8'h72;

8'h1f: c=8'hc0;

8'h20: c=8'hb7;

8'h21: c=8'hfd;

8'h22: c=8'h93;

8'h23: c=8'h26;

8'h24: c=8'h36;

8'h25: c=8'h3f;

8'h26: c=8'hf7;

8'h27: c=8'hcc;

8'h28: c=8'h34;

8'h29: c=8'ha5;

8'h2a: c=8'he5;

8'h2b: c=8'hf1;

8'h2c: c=8'h71;

8'h2d: c=8'hd8;

8'h2e: c=8'h31;

8'h2f: c=8'h15;

8'h30: c=8'h04;

8'h31: c=8'hc7;

8'h32: c=8'h23;

8'h33: c=8'hc3;

8'h34: c=8'h18;

8'h35: c=8'h96;

8'h36: c=8'h05;

8'h37: c=8'h9a;

8'h38: c=8'h07;

8'h39: c=8'h12;

8'h3a: c=8'h80;

8'h3b: c=8'he2;

8'h3c: c=8'heb;

8'h3d: c=8'h27;

8'h3e: c=8'hb2;

8'h3f: c=8'h75;

8'h40: c=8'h09;

8'h41: c=8'h83;

8'h42: c=8'h2c;

8'h43: c=8'h1a;

8'h44: c=8'h1b;

8'h45: c=8'h6e;

8'h46: c=8'h5a;

8'h47: c=8'ha0;

8'h48: c=8'h52;

8'h49: c=8'h3b;

8'h4a: c=8'hd6;

8'h4b: c=8'hb3;

8'h4c: c=8'h29;

8'h4d: c=8'he3;

8'h4e: c=8'h2f;

8'h4f: c=8'h84;

8'h50: c=8'h53;

8'h51: c=8'hd1;

8'h52: c=8'h00;

8'h53: c=8'hed;

8'h54: c=8'h20;

8'h55: c=8'hfc;

8'h56: c=8'hb1;

8'h57: c=8'h5b;

8'h58: c=8'h6a;

8'h59: c=8'hcb;

8'h5a: c=8'hbe;

8'h5b: c=8'h39;

8'h5c: c=8'h4a;

8'h5d: c=8'h4c;

8'h5e: c=8'h58;

8'h5f: c=8'hcf;

8'h60: c=8'hd0;

8'h61: c=8'hef;

8'h62: c=8'haa;

8'h63: c=8'hfb;

8'h64: c=8'h43;

8'h65: c=8'h4d;

8'h66: c=8'h33;

8'h67: c=8'h85;

8'h68: c=8'h45;

8'h69: c=8'hf9;

8'h6a: c=8'h02;

8'h6b: c=8'h7f;

8'h6c: c=8'h50;

8'h6d: c=8'h3c;

8'h6e: c=8'h9f;

8'h6f: c=8'ha8;

8'h70: c=8'h51;

8'h71: c=8'ha3;

8'h72: c=8'h40;

8'h73: c=8'h8f;

8'h74: c=8'h92;

8'h75: c=8'h9d;

8'h76: c=8'h38;

8'h77: c=8'hf5;

8'h78: c=8'hbc;

8'h79: c=8'hb6;

8'h7a: c=8'hda;

8'h7b: c=8'h21;

8'h7c: c=8'h10;

8'h7d: c=8'hff;

8'h7e: c=8'hf3;

8'h7f: c=8'hd2;

8'h80: c=8'hcd;

8'h81: c=8'h0c;

8'h82: c=8'h13;

8'h83: c=8'hec;

8'h84: c=8'h5f;

8'h85: c=8'h97;

8'h86: c=8'h44;

8'h87: c=8'h17;

8'h88: c=8'hc4;

8'h89: c=8'ha7;

8'h8a: c=8'h7e;

8'h8b: c=8'h3d;

8'h8c: c=8'h64;

8'h8d: c=8'h5d;

8'h8e: c=8'h19;

8'h8f: c=8'h73;

8'h90: c=8'h60;

8'h91: c=8'h81;

8'h92: c=8'h4f;

8'h93: c=8'hdc;

8'h94: c=8'h22;

8'h95: c=8'h2a;

8'h96: c=8'h90;

8'h97: c=8'h88;

8'h98: c=8'h46;

8'h99: c=8'hee;

8'h9a: c=8'hb8;

8'h9b: c=8'h14;

8'h9c: c=8'hde;

8'h9d: c=8'h5e;

8'h9e: c=8'h0b;

8'h9f: c=8'hdb;

8'ha0: c=8'he0;

8'ha1: c=8'h32;

8'ha2: c=8'h3a;

8'ha3: c=8'h0a;

8'ha4: c=8'h49;

8'ha5: c=8'h06;

8'ha6: c=8'h24;

8'ha7: c=8'h5c;

8'ha8: c=8'hc2;

8'ha9: c=8'hd3;

8'haa: c=8'hac;

8'hab: c=8'h62;

8'hac: c=8'h91;

8'had: c=8'h95;

8'hae: c=8'he4;

8'haf: c=8'h79;

8'hb0: c=8'he7;

8'hb1: c=8'hc8;

8'hb2: c=8'h37;

8'hb3: c=8'h6d;

8'hb4: c=8'h8d;

8'hb5: c=8'hd5;

8'hb6: c=8'h4e;

8'hb7: c=8'ha9;

8'hb8: c=8'h6c;

8'hb9: c=8'h56;

8'hba: c=8'hf4;

8'hbb: c=8'hea;

8'hbc: c=8'h65;

8'hbd: c=8'h7a;

8'hbe: c=8'hae;

8'hbf: c=8'h08;

8'hc0: c=8'hba;

8'hc1: c=8'h78;

8'hc2: c=8'h25;

8'hc3: c=8'h2e;

8'hc4: c=8'h1c;

8'hc5: c=8'ha6;

8'hc6: c=8'hb4;

8'hc7: c=8'hc6;

8'hc8: c=8'he8;

8'hc9: c=8'hdd;

8'hca: c=8'h74;

8'hcb: c=8'h1f;

8'hcc: c=8'h4b;

8'hcd: c=8'hbd;

8'hce: c=8'h8b;

8'hcf: c=8'h8a;

8'hd0: c=8'h70;

8'hd1: c=8'h3e;

8'hd2: c=8'hb5;

8'hd3: c=8'h66;

8'hd4: c=8'h48;

8'hd5: c=8'h03;

8'hd6: c=8'hf6;

8'hd7: c=8'h0e;

8'hd8: c=8'h61;

8'hd9: c=8'h35;

8'hda: c=8'h57;

8'hdb: c=8'hb9;

8'hdc: c=8'h86;

8'hdd: c=8'hc1;

8'hde: c=8'h1d;

8'hdf: c=8'h9e;

8'he0: c=8'he1;

8'he1: c=8'hf8;

8'he2: c=8'h98;

8'he3: c=8'h11;

8'he4: c=8'h69;

8'he5: c=8'hd9;

8'he6: c=8'h8e;

8'he7: c=8'h94;

8'he8: c=8'h9b;

8'he9: c=8'h1e;

8'hea: c=8'h87;

8'heb: c=8'he9;

8'hec: c=8'hce;

8'hed: c=8'h55;

8'hee: c=8'h28;

8'hef: c=8'hdf;

8'hf0: c=8'h8c;

8'hf1: c=8'ha1;

8'hf2: c=8'h89;

8'hf3: c=8'h0d;

8'hf4: c=8'hbf;

8'hf5: c=8'he6;

8'hf6: c=8'h42;

8'hf7: c=8'h68;

8'hf8: c=8'h41;

8'hf9: c=8'h99;

8'hfa: c=8'h2d;

8'hfb: c=8'h0f;

8'hfc: c=8'hb0;

8'hfd: c=8'h54;

8'hfe: c=8'hbb;

8'hff: c=8'h16;

endcase

endmodule

**DECRYPTION MODULE**

**// AES_decryption Module**

module AES_decryption

(

input [127:0] byteKey, byteText,

input clk,rst,enable,

output reg [127:0] resultantOutput,

output reg fetchAll,initialStep

);

integer i,j,y;

reg [1:0] loop1;

reg [1:0] loop2;

reg [127:0] key, text;

reg initiateRotating,initiateShiftingRows,initiateMixingColumns,kyIntiation;

wire startRotating,shiftingStart,mixingColumnsStart;

reg finish;

reg [127:0] sendTextInput,receiveTextOutput;

reg [127:0] sendKeyInput;

wire [127:0] roundingTextStart,TextSubbytingStart,textShiftingRows;

wire [127:0] textMixingColumns;

reg [3:0] keyNum;

reg taskComplete;

reg [127:0] kTransferO [10:0];

integer ro, flag;

reg [7:0] a1;

reg [7:0] a2;

reg [7:0] a3;

reg [7:0] a4;

reg [7:0] c;

reg [31:0] rcon [10:0];

reg [31:0] ladder1 ;

reg [31:0] ladder2 ;

task getResult;

input [7:0] z;

case (z) // Sbox values for byte substitution during key expansion

8'h00: c=8'h63;

8'h01: c=8'h7c;

8'h02: c=8'h77;

8'h03: c=8'h7b;

8'h04: c=8'hf2;

8'h05: c=8'h6b;

8'h06: c=8'h6f;

8'h07: c=8'hc5;

8'h08: c=8'h30;

8'h09: c=8'h01;

8'h0a: c=8'h67;

8'h0b: c=8'h2b;

8'h0c: c=8'hfe;

8'h0d: c=8'hd7;

8'h0e: c=8'hab;

8'h0f: c=8'h76;

8'h10: c=8'hca;

8'h11: c=8'h82;

8'h12: c=8'hc9;

8'h13: c=8'h7d;

8'h14: c=8'hfa;

8'h15: c=8'h59;

8'h16: c=8'h47;

8'h17: c=8'hf0;

8'h18: c=8'had;

8'h19: c=8'hd4;

8'h1a: c=8'ha2;

8'h1b: c=8'haf;

8'h1c: c=8'h9c;

8'h1d: c=8'ha4;

8'h1e: c=8'h72;

8'h1f: c=8'hc0;

8'h20: c=8'hb7;

8'h21: c=8'hfd;

8'h22: c=8'h93;

8'h23: c=8'h26;

8'h24: c=8'h36;

8'h25: c=8'h3f;

8'h26: c=8'hf7;

8'h27: c=8'hcc;

8'h28: c=8'h34;

8'h29: c=8'ha5;

8'h2a: c=8'he5;

8'h2b: c=8'hf1;

8'h2c: c=8'h71;

8'h2d: c=8'hd8;

8'h2e: c=8'h31;

8'h2f: c=8'h15;

8'h30: c=8'h04;

8'h31: c=8'hc7;

8'h32: c=8'h23;

8'h33: c=8'hc3;

8'h34: c=8'h18;

8'h35: c=8'h96;

8'h36: c=8'h05;

8'h37: c=8'h9a;

8'h38: c=8'h07;

8'h39: c=8'h12;

8'h3a: c=8'h80;

8'h3b: c=8'he2;

8'h3c: c=8'heb;

8'h3d: c=8'h27;

8'h3e: c=8'hb2;

8'h3f: c=8'h75;

8'h40: c=8'h09;

8'h41: c=8'h83;

8'h42: c=8'h2c;

8'h43: c=8'h1a;

8'h44: c=8'h1b;

8'h45: c=8'h6e;

8'h46: c=8'h5a;

8'h47: c=8'ha0;

8'h48: c=8'h52;

8'h49: c=8'h3b;

8'h4a: c=8'hd6;

8'h4b: c=8'hb3;

8'h4c: c=8'h29;

8'h4d: c=8'he3;

8'h4e: c=8'h2f;

8'h4f: c=8'h84;

8'h50: c=8'h53;

8'h51: c=8'hd1;

8'h52: c=8'h00;

8'h53: c=8'hed;

8'h54: c=8'h20;

8'h55: c=8'hfc;

8'h56: c=8'hb1;

8'h57: c=8'h5b;

8'h58: c=8'h6a;

8'h59: c=8'hcb;

8'h5a: c=8'hbe;

8'h5b: c=8'h39;

8'h5c: c=8'h4a;

8'h5d: c=8'h4c;

8'h5e: c=8'h58;

8'h5f: c=8'hcf;

8'h60: c=8'hd0;

8'h61: c=8'hef;

8'h62: c=8'haa;

8'h63: c=8'hfb;

8'h64: c=8'h43;

8'h65: c=8'h4d;

8'h66: c=8'h33;

8'h67: c=8'h85;

8'h68: c=8'h45;

8'h69: c=8'hf9;

8'h6a: c=8'h02;

8'h6b: c=8'h7f;

8'h6c: c=8'h50;

8'h6d: c=8'h3c;

8'h6e: c=8'h9f;

8'h6f: c=8'ha8;

8'h70: c=8'h51;

8'h71: c=8'ha3;

8'h72: c=8'h40;

8'h73: c=8'h8f;

8'h74: c=8'h92;

8'h75: c=8'h9d;

8'h76: c=8'h38;

8'h77: c=8'hf5;

8'h78: c=8'hbc;

8'h79: c=8'hb6;

8'h7a: c=8'hda;

8'h7b: c=8'h21;

8'h7c: c=8'h10;

8'h7d: c=8'hff;

8'h7e: c=8'hf3;

8'h7f: c=8'hd2;

8'h80: c=8'hcd;

8'h81: c=8'h0c;

8'h82: c=8'h13;

8'h83: c=8'hec;

8'h84: c=8'h5f;

8'h85: c=8'h97;

8'h86: c=8'h44;

8'h87: c=8'h17;

8'h88: c=8'hc4;

8'h89: c=8'ha7;

8'h8a: c=8'h7e;

8'h8b: c=8'h3d;

8'h8c: c=8'h64;

8'h8d: c=8'h5d;

8'h8e: c=8'h19;

8'h8f: c=8'h73;

8'h90: c=8'h60;

8'h91: c=8'h81;

8'h92: c=8'h4f;

8'h93: c=8'hdc;

8'h94: c=8'h22;

8'h95: c=8'h2a;

8'h96: c=8'h90;

8'h97: c=8'h88;

8'h98: c=8'h46;

8'h99: c=8'hee;

8'h9a: c=8'hb8;

8'h9b: c=8'h14;

8'h9c: c=8'hde;

8'h9d: c=8'h5e;

8'h9e: c=8'h0b;

8'h9f: c=8'hdb;

8'ha0: c=8'he0;

8'ha1: c=8'h32;

8'ha2: c=8'h3a;

8'ha3: c=8'h0a;

8'ha4: c=8'h49;

8'ha5: c=8'h06;

8'ha6: c=8'h24;

8'ha7: c=8'h5c;

8'ha8: c=8'hc2;

8'ha9: c=8'hd3;

8'haa: c=8'hac;

8'hab: c=8'h62;

8'hac: c=8'h91;

8'had: c=8'h95;

8'hae: c=8'he4;

8'haf: c=8'h79;

8'hb0: c=8'he7;

8'hb1: c=8'hc8;

8'hb2: c=8'h37;

8'hb3: c=8'h6d;

8'hb4: c=8'h8d;

8'hb5: c=8'hd5;

8'hb6: c=8'h4e;

8'hb7: c=8'ha9;

8'hb8: c=8'h6c;

8'hb9: c=8'h56;

8'hba: c=8'hf4;

8'hbb: c=8'hea;

8'hbc: c=8'h65;

8'hbd: c=8'h7a;

8'hbe: c=8'hae;

8'hbf: c=8'h08;

8'hc0: c=8'hba;

8'hc1: c=8'h78;

8'hc2: c=8'h25;

8'hc3: c=8'h2e;

8'hc4: c=8'h1c;

8'hc5: c=8'ha6;

8'hc6: c=8'hb4;

8'hc7: c=8'hc6;

8'hc8: c=8'he8;

8'hc9: c=8'hdd;

8'hca: c=8'h74;

8'hcb: c=8'h1f;

8'hcc: c=8'h4b;

8'hcd: c=8'hbd;

8'hce: c=8'h8b;

8'hcf: c=8'h8a;

8'hd0: c=8'h70;

8'hd1: c=8'h3e;

8'hd2: c=8'hb5;

8'hd3: c=8'h66;

8'hd4: c=8'h48;

8'hd5: c=8'h03;

8'hd6: c=8'hf6;

8'hd7: c=8'h0e;

8'hd8: c=8'h61;

8'hd9: c=8'h35;

8'hda: c=8'h57;

8'hdb: c=8'hb9;

8'hdc: c=8'h86;

8'hdd: c=8'hc1;

8'hde: c=8'h1d;

8'hdf: c=8'h9e;

8'he0: c=8'he1;

8'he1: c=8'hf8;

8'he2: c=8'h98;

8'he3: c=8'h11;

8'he4: c=8'h69;

8'he5: c=8'hd9;

8'he6: c=8'h8e;

8'he7: c=8'h94;

8'he8: c=8'h9b;

8'he9: c=8'h1e;

8'hea: c=8'h87;

8'heb: c=8'he9;

8'hec: c=8'hce;

8'hed: c=8'h55;

8'hee: c=8'h28;

8'hef: c=8'hdf;

8'hf0: c=8'h8c;

8'hf1: c=8'ha1;

8'hf2: c=8'h89;

8'hf3: c=8'h0d;

8'hf4: c=8'hbf;

8'hf5: c=8'he6;

8'hf6: c=8'h42;

8'hf7: c=8'h68;

8'hf8: c=8'h41;

8'hf9: c=8'h99;

8'hfa: c=8'h2d;

8'hfb: c=8'h0f;

8'hfc: c=8'hb0;

8'hfd: c=8'h54;

8'hfe: c=8'hbb;

8'hff: c=8'h16;

endcase

endtask

AddRoundKey S(.inputkey(sendKeyInput),.inputState(sendTextInput),.clk(clk),.reset(rst),.enable(initiateRotating),.inputOut(roundingTextStart),.success(startRotating));

genvar itr;

generate

for (itr = 0 ; itr <= 127; itr = itr+32)begin : block_subbyte

InvSubByte statSub (.data(textShiftingRows[itr +:32]) , .result(TextSubbytingStart[itr +:32]));

end

endgenerate

InvShiftRows Sft (.enable(initiateShiftingRows),.clk(clk),.reset(rst),.data(sendTextInput),.rotatedValue(textShiftingRows),.success(shiftingStart) );

InvMixColumns Mi (.value(sendTextInput), .clk (clk), .enable(initiateMixingColumns), .reset(rst), .outValue(textMixingColumns), .success(mixingColumnsStart));

always @(posedge clk)begin

if (rst) begin

taskComplete <= 0;

key<=128'd0;

text<=128'd0;

fetchAll<=1'd0;

initialStep<=1'd0;

i <= 1;

resultantOutput <= 128'h00000000000000000000000000000000;

sendTextInput <= 128'h00000000000000000000000000000000;

receiveTextOutput <= 128'h00000000000000000000000000000000;

j <= 1;

loop2 <= 0 ;

end

else if (enable)begin

case(loop2)

0:

begin

loop2 <= 1;

i <= 1;

end

1:

begin

if (i>0) begin

fetchAll<=1'd1;

taskComplete <= 0;

text<=byteText; // Initializewith the ciphertext

i<=i-1;

end

else begin

fetchAll<=1'd0;

taskComplete <= 1;

loop2 <= 2;

end

end

2: begin

if(finish) begin

initialStep <= 0;

loop2 <= 3;

j <= 1;

end else initialStep <= 0;

end

3: begin

if ( j > 0) begin

initialStep <= 1;

resultantOutput <= receiveTextOutput; // Resultant plaintext

j <= j - 1;

end

else begin

initialStep <= 0;

loop2 <= 0;

end

end

endcase

end else loop2 <= 0;

if (rst)

begin

keyNum <= 0;

loop1 <= 0;

initiateMixingColumns <= 0;

kyIntiation <= 0;

initiateRotating <= 0;

initiateShiftingRows <= 0;

receiveTextOutput <= 0;

finish <= 0;

end

else if( (enable == 1 ) && (taskComplete == 1))

begin

if ( keyNum <= 11 ) begin

case (loop1)

2'b00:

begin

// Add round key

ro = 10;

kTransferO[0][127:0] = byteKey;

kTransferO[1][127:0] = 128'b0;

kTransferO[2][127:0] = 128'b0;

kTransferO[3][127:0] = 128'b0;

kTransferO[4][127:0] = 128'b0;

kTransferO[5][127:0] = 128'b0;

kTransferO[6][127:0] = 128'b0;

kTransferO[7][127:0] = 128'b0;

kTransferO[8][127:0] = 128'b0;

kTransferO[9][127:0] = 128'b0;

kTransferO[10][127:0] = 128'b0;

rcon[1][31:0] = 32'h01000000;

rcon[2][31:0] = 32'h02000000;

rcon[3][31:0] = 32'h04000000;

rcon[4][31:0] = 32'h08000000;

rcon[5][31:0] = 32'h10000000;

rcon[6][31:0] = 32'h20000000;

rcon[7][31:0] = 32'h40000000;

rcon[8][31:0] = 32'h80000000;

rcon[9][31:0] = 32'h1b000000;

rcon[10][31:0] = 32'h36000000;

for (y = 1; y <= 10; y = y + 1) begin //Start of key expansion

ladder1 = { kTransferO[y-1][23:0] , kTransferO[y-1][31:24] };

getResult(ladder1[23:16]);

a1 = c;

getResult(ladder1[15:8]);

a2 = c;

getResult(ladder1[7:0]);

a3 = c;

getResult(ladder1[31:24]);

a4 = c;

ladder2 = { a4, a1, a2, a3 };

kTransferO[y][127:96] = kTransferO[y-1][127:96] ^ ladder2 ^ rcon[y];

kTransferO[y][95:64] = kTransferO[y-1][95:64] ^ kTransferO[y][127:96];

kTransferO[y][63:32] = kTransferO[y-1][63:32] ^ kTransferO[y][95:64];

kTransferO[y][31:0] = kTransferO[y-1][31:0] ^ kTransferO[y][63:32];

end // End of key expansion

sendKeyInput = kTransferO[ro];

sendTextInput <= text;

keyNum = 4'h1;

initiateRotating = 1;

ro = ro - 1;

loop1 = 2'b01;

flag = 1;

finish = 0;

end

2'b01:

begin

//Inverse shift rows

if ((startRotating == 1) || (mixingColumnsStart == 1)) begin

if((keyNum <= 10) && (flag == 0))

sendTextInput <= textMixingColumns;

else

sendTextInput <= roundingTextStart;

sendKeyInput = kTransferO[ro];

initiateRotating = 0;

initiateShiftingRows = 1;

initiateMixingColumns = 0;

loop1 = 2'b10;

flag = 0;

end

finish <= 0;

end

2'b10:

begin

// Inverse sub bytes

if (shiftingStart == 1) begin

sendTextInput <= TextSubbytingStart;

initiateShiftingRows = 0;

initiateMixingColumns = 0;

initiateRotating = 1;

if (keyNum < 10)

loop1 = 2'b11;

else begin

keyNum = keyNum + 1;

loop1 = 2'b01;

end

if(keyNum == 11)

ro = ro - 1;

end

finish <= 0;

end

2'b11:

begin

//Inverse mix columns

if (startRotating == 1) begin

sendTextInput <= roundingTextStart;

initiateRotating = 0;

initiateShiftingRows = 0;

initiateMixingColumns = 1;

loop1 = 2'b01;

ro = ro - 1;

keyNum = keyNum + 1;

end

finish <= 0;

end

endcase

end

else begin

// When keyNum > 11

receiveTextOutput <= roundingTextStart;

finish <= 1;

keyNum <= 0;

loop1 <= 0;

initiateMixingColumns <= 0;

kyIntiation <= 0;

initiateRotating <= 0;

initiateShiftingRows <= 0;

end

end else

begin

keyNum <= 0;

loop1 <= 0;

initiateMixingColumns <= 0;

kyIntiation <= 0;

initiateRotating <= 0;

initiateShiftingRows <= 0;

receiveTextOutput <= 0;

finish <= 0;

end

end //End always

endmodule

**INVERSE SHIFT ROW MODULE**

**// InvShiftRows Module**

module InvShiftRows(

input enable,clk,reset,

input wire [0:127] data,

output reg [0:127] rotatedValue,

output reg success);

always @(negedge clk)

begin

if(reset)

begin

rotatedValue <= 128'h00000000000000000000000000000000;

success <= 0;

end

else if (enable) begin

//No shifting

rotatedValue[0+:8] <= data[0+:8];

rotatedValue[32+:8] <= data[32+:8];

rotatedValue[64+:8] <= data[64+:8];

rotatedValue[96+:8] <= data[96+:8];

//1 byte right shift

rotatedValue[8+:8] <= data[104+:8];

rotatedValue[40+:8] <= data[8+:8];

rotatedValue[72+:8] <= data[40+:8];

rotatedValue[104+:8] <= data[72+:8];

//2 byte right shift

rotatedValue[16+:8] <= data[80+:8];

rotatedValue[48+:8] <= data[112+:8];

rotatedValue[80+:8] <= data[16+:8];

rotatedValue[112+:8] <= data[48+:8];

//3 byte right shift

rotatedValue[24+:8] <= data[56+:8];

rotatedValue[56+:8] <= data[88+:8];

rotatedValue[88+:8] <= data[120+:8];

rotatedValue[120+:8] <= data[24+:8];

success <= 1;

end

else success <= 0;

end //End always

endmodule

**INVERSE SUBSTITUTION BYTE MODULE**

**// InvSubByte Module**

module InvSubByte(

input wire [31:0] data,

output wire [31:0] result

);

genvar i;

genvar j;

generate

for(i=0; i<=31; i=i+8) begin : generate_inversesbox_identifier

InvS_box isbox(data[i +:8] , result[i +:8]);

end

endgenerate

endmodule

**INVERSE MIX COLUMN MODULE**

**// InvMixColumns Module**

module InvMixColumns(

input wire [127:0] value,

input wire clk,enable,reset,

output reg [127:0] outValue,

output reg success);

function [7:0] MultiplyByTwo;

input [7:0] x;

begin

if(x[7] == 1) MultiplyByTwo = ((x << 1) ^ 8'h1b);

else MultiplyByTwo = x << 1;

end

endfunction

integer i;

always@(negedge clk) begin

if(reset) begin

outValue <= 128'h00000000000000000000000000000000;

i<= 0;

success <= 0;

end

else if(enable) begin

for(i=0;i<=3;i=i+1) begin

outValue[i*32+:8] <= MultiplyByTwo(MultiplyByTwo(MultiplyByTwo(value[(i*32)+:8]) ^ value[(i*32)+:8]) ^ value[(i*32)+:8]) ^ (MultiplyByTwo(MultiplyByTwo(MultiplyByTwo(value[(i*32 + 8)+:8]))) ^ value[(i*32 + 8)+:8]) ^ (MultiplyByTwo(MultiplyByTwo(MultiplyByTwo(value[(i*32 + 16)+:8]) ^ value[(i*32 + 16)+:8])) ^ value[(i*32 + 16)+:8]) ^ (MultiplyByTwo(MultiplyByTwo(MultiplyByTwo(value[(i*32 + 24)+:8])) ^ value[(i*32 + 24)+:8]) ^ value[(i*32 + 24)+:8]);

outValue[(i*32 + 8)+:8] <= (MultiplyByTwo(MultiplyByTwo(MultiplyByTwo(value[(i*32)+:8])) ^ value[(i*32)+:8]) ^ value[(i*32)+:8]) ^ MultiplyByTwo(MultiplyByTwo(MultiplyByTwo(value[(i*32 + 8)+:8]) ^ value[(i*32 + 8)+:8]) ^ value[(i*32 + 8)+:8]) ^ (MultiplyByTwo(MultiplyByTwo(MultiplyByTwo(value[(i*32 + 16)+:8]))) ^ value[(i*32 + 16)+:8]) ^ (MultiplyByTwo(MultiplyByTwo(MultiplyByTwo(value[(i*32 + 24)+:8]) ^ value[(i*32 + 24)+:8])) ^ value[(i*32 + 24)+:8]);

outValue[(i*32 + 16)+:8] <= (MultiplyByTwo(MultiplyByTwo(MultiplyByTwo(value[(i*32)+:8]) ^ value[(i*32)+:8])) ^ value[(i*32)+:8]) ^ (MultiplyByTwo(MultiplyByTwo(MultiplyByTwo(value[(i*32 + 8)+:8])) ^ value[(i*32 + 8)+:8]) ^ value[(i*32 + 8)+:8]) ^ MultiplyByTwo(MultiplyByTwo(MultiplyByTwo(value[(i*32 + 16)+:8]) ^ value[(i*32 + 16)+:8]) ^ value[(i*32 + 16)+:8]) ^ (MultiplyByTwo(MultiplyByTwo(MultiplyByTwo(value[(i*32 + 24)+:8]))) ^ value[(i*32 + 24)+:8]);

outValue[(i*32 + 24)+:8] <= (MultiplyByTwo(MultiplyByTwo(MultiplyByTwo(value[(i*32)+:8]))) ^ value[(i*32)+:8]) ^ (MultiplyByTwo(MultiplyByTwo(MultiplyByTwo(value[(i*32 + 8)+:8]) ^ value[(i*32 + 8)+:8])) ^ value[(i*32 + 8)+:8]) ^ (MultiplyByTwo(MultiplyByTwo(MultiplyByTwo(value[(i*32 + 16)+:8])) ^ value[(i*32 + 16)+:8]) ^ value[(i*32 + 16)+:8]) ^ MultiplyByTwo(MultiplyByTwo(MultiplyByTwo(value[(i*32 + 24)+:8]) ^ value[(i*32 + 24)+:8]) ^ value[(i*32 + 24)+:8]);

end

success <= 1;

end else success <= 0;

end //End always

endmodule

**INVERSE SBOX MODULE**

**// InvS_box Module**

module InvS_box(input [7:0] a,output reg [7:0] c);

always @ ( a )

case (a)

8'h00: c=8'h52;

8'h01: c=8'h09;

8'h02: c=8'h6a;

8'h03: c=8'hd5;

8'h04: c=8'h30;

8'h05: c=8'h36;

8'h06: c=8'ha5;

8'h07: c=8'h38;

8'h08: c=8'hbf;

8'h09: c=8'h40;

8'h0a: c=8'ha3;

8'h0b: c=8'h9e;

8'h0c: c=8'h81;

8'h0d: c=8'hf3;

8'h0e: c=8'hd7;

8'h0f: c=8'hfb;

8'h10: c=8'h7c;

8'h11: c=8'he3;

8'h12: c=8'h39;

8'h13: c=8'h82;

8'h14: c=8'h9b;

8'h15: c=8'h2f;

8'h16: c=8'hff;

8'h17: c=8'h87;

8'h18: c=8'h34;

8'h19: c=8'h8e;

8'h1a: c=8'h43;

8'h1b: c=8'h44;

8'h1c: c=8'hc4;

8'h1d: c=8'hde;

8'h1e: c=8'he9;

8'h1f: c=8'hcb;

8'h20: c=8'h54;

8'h21: c=8'h7b;

8'h22: c=8'h94;

8'h23: c=8'h32;

8'h24: c=8'ha6;

8'h25: c=8'hc2;

8'h26: c=8'h23;

8'h27: c=8'h3d;

8'h28: c=8'hee;

8'h29: c=8'h4c;

8'h2a: c=8'h95;

8'h2b: c=8'h0b;

8'h2c: c=8'h42;

8'h2d: c=8'hfa;

8'h2e: c=8'hc3;

8'h2f: c=8'h4e;

8'h30: c=8'h08;

8'h31: c=8'h2e;

8'h32: c=8'ha1;

8'h33: c=8'h66;

8'h34: c=8'h28;

8'h35: c=8'hd9;

8'h36: c=8'h24;

8'h37: c=8'hb2;

8'h38: c=8'h76;

8'h39: c=8'h5b;

8'h3a: c=8'ha2;

8'h3b: c=8'h49;

8'h3c: c=8'h6d;

8'h3d: c=8'h8b;

8'h3e: c=8'hd1;

8'h3f: c=8'h25;

8'h40: c=8'h72;

8'h41: c=8'hf8;

8'h42: c=8'hf6;

8'h43: c=8'h64;

8'h44: c=8'h86;

8'h45: c=8'h68;

8'h46: c=8'h98;

8'h47: c=8'h16;

8'h48: c=8'hd4;

8'h49: c=8'ha4;

8'h4a: c=8'h5c;

8'h4b: c=8'hcc;

8'h4c: c=8'h5d;

8'h4d: c=8'h65;

8'h4e: c=8'hb6;

8'h4f: c=8'h92;

8'h50: c=8'h6c;

8'h51: c=8'h70;

8'h52: c=8'h48;

8'h53: c=8'h50;

8'h54: c=8'hfd;

8'h55: c=8'hed;

8'h56: c=8'hb9;

8'h57: c=8'hda;

8'h58: c=8'h5e;

8'h59: c=8'h15;

8'h5a: c=8'h46;

8'h5b: c=8'h57;

8'h5c: c=8'ha7;

8'h5d: c=8'h8d;

8'h5e: c=8'h9d;

8'h5f: c=8'h84;

8'h60: c=8'h90;

8'h61: c=8'hd8;

8'h62: c=8'hab;

8'h63: c=8'h00;

8'h64: c=8'h8c;

8'h65: c=8'hbc;

8'h66: c=8'hd3;

8'h67: c=8'h0a;

8'h68: c=8'hf7;

8'h69: c=8'he4;

8'h6a: c=8'h58;

8'h6b: c=8'h05;

8'h6c: c=8'hb8;

8'h6d: c=8'hb3;

8'h6e: c=8'h45;

8'h6f: c=8'h06;

8'h70: c=8'hd0;

8'h71: c=8'h2c;

8'h72: c=8'h1e;

8'h73: c=8'h8f;

8'h74: c=8'hca;

8'h75: c=8'h3f;

8'h76: c=8'h0f;

8'h77: c=8'h02;

8'h78: c=8'hc1;

8'h79: c=8'haf;

8'h7a: c=8'hbd;

8'h7b: c=8'h03;

8'h7c: c=8'h01;

8'h7d: c=8'h13;

8'h7e: c=8'h8a;

8'h7f: c=8'h6b;

8'h80: c=8'h3a;

8'h81: c=8'h91;

8'h82: c=8'h11;

8'h83: c=8'h41;

8'h84: c=8'h4f;

8'h85: c=8'h67;

8'h86: c=8'hdc;

8'h87: c=8'hea;

8'h88: c=8'h97;

8'h89: c=8'hf2;

8'h8a: c=8'hcf;

8'h8b: c=8'hce;

8'h8c: c=8'hf0;

8'h8d: c=8'hb4;

8'h8e: c=8'he6;

8'h8f: c=8'h73;

8'h90: c=8'h96;

8'h91: c=8'hac;

8'h92: c=8'h74;

8'h93: c=8'h22;

8'h94: c=8'he7;

8'h95: c=8'had;

8'h96: c=8'h35;

8'h97: c=8'h85;

8'h98: c=8'he2;

8'h99: c=8'hf9;

8'h9a: c=8'h37;

8'h9b: c=8'he8;

8'h9c: c=8'h1c;

8'h9d: c=8'h75;

8'h9e: c=8'hdf;

8'h9f: c=8'h6e;

8'ha0: c=8'h47;

8'ha1: c=8'hf1;

8'ha2: c=8'h1a;

8'ha3: c=8'h71;

8'ha4: c=8'h1d;

8'ha5: c=8'h29;

8'ha6: c=8'hc5;

8'ha7: c=8'h89;

8'ha8: c=8'h6f;

8'ha9: c=8'hb7;

8'haa: c=8'h62;

8'hab: c=8'h0e;

8'hac: c=8'haa;

8'had: c=8'h18;

8'hae: c=8'hbe;

8'haf: c=8'h1b;

8'hb0: c=8'hfc;

8'hb1: c=8'h56;

8'hb2: c=8'h3e;

8'hb3: c=8'h4b;

8'hb4: c=8'hc6;

8'hb5: c=8'hd2;

8'hb6: c=8'h79;

8'hb7: c=8'h20;

8'hb8: c=8'h9a;

8'hb9: c=8'hdb;

8'hba: c=8'hc0;

8'hbb: c=8'hfe;

8'hbc: c=8'h78;

8'hbd: c=8'hcd;

8'hbe: c=8'h5a;

8'hbf: c=8'hf4;

8'hc0: c=8'h1f;

8'hc1: c=8'hdd;

8'hc2: c=8'ha8;

8'hc3: c=8'h33;

8'hc4: c=8'h88;

8'hc5: c=8'h07;

8'hc6: c=8'hc7;

8'hc7: c=8'h31;

8'hc8: c=8'hb1;

8'hc9: c=8'h12;

8'hca: c=8'h10;

8'hcb: c=8'h59;

8'hcc: c=8'h27;

8'hcd: c=8'h80;

8'hce: c=8'hec;

8'hcf: c=8'h5f;

8'hd0: c=8'h60;

8'hd1: c=8'h51;

8'hd2: c=8'h7f;

8'hd3: c=8'ha9;

8'hd4: c=8'h19;

8'hd5: c=8'hb5;

8'hd6: c=8'h4a;

8'hd7: c=8'h0d;

8'hd8: c=8'h2d;

8'hd9: c=8'he5;

8'hda: c=8'h7a;

8'hdb: c=8'h9f;

8'hdc: c=8'h93;

8'hdd: c=8'hc9;

8'hde: c=8'h9c;

8'hdf: c=8'hef;

8'he0: c=8'ha0;

8'he1: c=8'he0;

8'he2: c=8'h3b;

8'he3: c=8'h4d;

8'he4: c=8'hae;

8'he5: c=8'h2a;

8'he6: c=8'hf5;

8'he7: c=8'hb0;

8'he8: c=8'hc8;

8'he9: c=8'heb;

8'hea: c=8'hbb;

8'heb: c=8'h3c;

8'hec: c=8'h83;

8'hed: c=8'h53;

8'hee: c=8'h99;

8'hef: c=8'h61;

8'hf0: c=8'h17;

8'hf1: c=8'h2b;

8'hf2: c=8'h04;

8'hf3: c=8'h7e;

8'hf4: c=8'hba;

8'hf5: c=8'h77;

8'hf6: c=8'hd6;

8'hf7: c=8'h26;

8'hf8: c=8'he1;

8'hf9: c=8'h69;

8'hfa: c=8'h14;

8'hfb: c=8'h63;

8'hfc: c=8'h55;

8'hfd: c=8'h21;

8'hfe: c=8'h0c;

8'hff: c=8'h7d;

endcase

endmodule

**OUTPUT RESPONSE ANALYZER MODULE**

**// ORA Module**

module ORA(input wire clk, input wire rst, input wire oraEnable, input [127:0] valueToXor, output reg[31:0] valueO,output reg ready);

integer i,j,k;

reg flag=0;

reg[31:0] tempValue;

reg [127:0] holdVlaue;

always @(posedge clk)begin : break_block

if(j==62)begin

disable break_block;

end

if(flag==0)begin

j=0;

tempValue = 32'b00000000000000000000000000000000;

flag=1;

end

else if(oraEnable==1)begin

if(holdVlaue!=valueToXor)begin

for(i=0;i<128;i=i+1)begin /* Linear-feedback shift register (LFSR) to genrate the expected candidate signature,which is later used to match with the Golden signature */

tempValue = {(valueToXor[i] ^ tempValue[31] ^ tempValue[25] ^ tempValue[22] ^ tempValue[21] ^ tempValue[15] ^ tempValue[11] ^ tempValue[10] ^ tempValue[9] ^ tempValue[7] ^ tempValue[6] ^ tempValue[4] ^ tempValue[3] ^ tempValue[1] ^ tempValue[0]), tempValue[31:1]};

end

holdVlaue = valueToXor;

end

valueO=tempValue;

j=j+1;

end

end //End always

endmodule
